# Type 1 interferon signature in peripheral blood mononuclear cells and monocytes of idiopathic inflammatory myopathy patients with different myositis-specific autoantibodies

**DOI:** 10.3389/fimmu.2023.1169057

**Published:** 2023-05-09

**Authors:** Mengdi Li, Yusheng Zhang, Wenzhe Zhang, Jinlei Sun, Rui Liu, Zhou Pan, Panpan Zhang, Shengyun Liu

**Affiliations:** ^1^ Department of Rheumatology and Clinical Immunology, The First Affiliated Hospital of Zhengzhou University, Zhengzhou, China; ^2^ Department of Radiology, The Third Affiliated Hospital of Zhengzhou University, Zhengzhou, China

**Keywords:** idiopathic inflammatory myopathy, anti-MDA5+ dermatomyositis, interferon, monocytes, RNA-sequencing

## Abstract

**Background:**

Myositis-specific autoantibodies (MSAs) are clinically used to diagnose and define idiopathic inflammatory myopathy (IIM) subsets. However, the underlying pathogenic mechanisms of patients with different MSAs remain unclear.

**Methods:**

A total of 158 Chinese patients with IIM and 167 gender- and age-matched healthy controls (HCs) were enrolled. Transcriptome sequencing (RNA-Seq) was performed with peripheral blood mononuclear cells (PBMCs), followed by the identification of differentially expressed genes (DEGs) and analysis of gene set enrichment analysis, immune cell infiltration, and WGCNA. Monocyte subsets and related cytokines/chemokines were quantified. The expressions of interferon (IFN)-related genes were validated using qRT-PCR and Western blot in both PBMCs and monocytes. We also performed correlation analysis and ROC analysis to explore the potential clinical significance of the IFN-related genes.

**Results:**

There were 1,364 genes altered in patients with IIM, including 952 upregulated and 412 downregulated genes. The type I interferon (IFN-I) pathway was remarkably activated in patients with IIM. Compared with patients with other MSAs, IFN-I signatures were significantly activated in patients with anti-melanoma differentiation-associated gene 5 (MDA5) antibodies. In total, 1,288 hub genes associated with IIM onset were identified using WGCNA, including 29 key DEGs associated with IFN signaling. The patients had more CD14brightCD16- classical, CD14brightCD16+ intermediate, and fewer CD14dimCD16+ non-classical monocyte subsets. Plasma cytokines like IL-6 and TNF and chemokines including CCL3 and MCPs increased. The validation of IFN-I-related gene expressions was consistent with the findings from RNA-Seq. The IFN-related genes were correlated with laboratory parameters and helpful for IIM diagnosis.

**Conclusion:**

Gene expressions were remarkably altered in the PBMCs of IIM patients. Anti-MDA5+ IIM patients had a more pronounced activated IFN signature than others. Monocytes exhibited a proinflammatory feature and contributed to the IFN signature of IIM patients.

## 1 Introduction

Idiopathic inflammatory myopathies (IIM) are a group of rare autoimmune diseases mainly affecting the skeletal muscles and other organs (1). Myositis-specific antibodies (MSAs), defined as autoantibodies found specifically in IIM patients, have been found to be strongly correlated with clinical manifestations and prognosis (2). Autoantibody specificities correlate with clinical features, such as the associations between anti-TIF1γ antibodies and high malignancy risk, and anti-melanoma differentiation-associated gene 5 (MDA5) antibodies and rapidly progressive interstitial lung disease (ILD) (3, 4). Muscle gene expression profiles, metabolic signatures, and pathways in plasma and urine samples were found different in MSA-typed IIM patients (5, 6). Compared with conventional subgrouping based on clinical phenotypes, autoantibody profiles perform better in knowing the immune mechanisms in IIM (7). Nowadays, the pathogenesis of IIM is not fully understood, so studies that are focused on different MSA-typed patients may provide valuable clues.

Hypotheses on the pathogenesis of IIM have been proposed as endoplasmic reticulum stress, vasculopathy, acquired interferonopathy, and so on (8–10). In the blood, skin, and muscles of IIM, upregulation of the IFN pathway has been verified and can reflect disease activity (11–13). Previous studies have highlighted the role of the adaptive immune system, especially T cells and B cells, in the immunopathogenesis of IIM (7, 14, 15). There is also a few evidence suggesting the involvement of monocyte/macrophage in IIM. Macrophage infiltration has been found in the muscles of all types of IIM, which is responsible for antigen presenting, necrotic muscle fiber invasion and elimination, and cytokine and chemokine production of IFN, IL-6, TNF-α, and so on (15–17). The activation and distribution pattern of monocytes/macrophages differs in the muscles of dermatomyositis (DM) and polymyositis (PM), and the macrophage infiltration mode relates to the serological subtypes (15, 18, 19). Activated monocytes/macrophages might be responsible for the cytokine storm in anti-MDA5-associated ILD (20). Aberrant mitochondrial biology in juvenile dermatomyositis (JDM) monocytes stimulates the expression of IFN-stimulated genes (ISGs) (21). Taken together, monocyte/macrophage may play a role in IIM pathogenesis.

The transcriptomic profile of peripheral blood mononuclear cells (PBMCs) of IIM patients needs to be further explored, especially for patients with distinct serological features. Therefore, we performed RNA-seq, bioinformatic analysis, and experimental validations in PBMCs to investigate immune cell and gene expression alterations in patients with different MSAs.

## 2 Materials and methods

### 2.1 Patient enrollment

A total of 158 IIM patients were recruited between October 2021 and October 2022 in the First Affiliated Hospital of Zhengzhou University (China). All patients were over 18 years old at the disease onset and met the Bohan and Peter criteria (22). Patients with other concurrent autoimmune diseases were excluded. This study was authorized by the Ethics Committee of Zhengzhou University’s First Affiliated Hospital (KY-2021-00805), and all participants provided written informed consent.

Anti-MDA5 and anti-TIF1γ antibodies were assayed using enzyme-linked immunosorbent assay (MBL, Japan). Anti-Jo-1, anti-EJ, anti-PL-7, anti-PL-12, anti-SRP, anti-Ro52, anti-PM-Scl 75, anti-PM-Scl 100, anti-Ku, and anti-Mi-2 antibodies were detected using line immunoassays (EUROIMMUN, Germany). For RNA-seq, 23 IIM patients who were newly diagnosed or in active disease status as well as eight gender- and age-matched healthy controls (HCs) were enrolled. Among these 23 patients, nine were anti-MDA5+, four were anti-Jo-1+, five were anti-TIF1γ+, and five were MSAs-. For flow cytometry, PBMCs were obtained from 40 IIM patients [anti-MDA5+ (*n* = 12), MSAs- (*n* = 10), anti-Jo-1+ (*n* = 7), anti-TIF1γ+ (*n* = 5), anti-EJ+ (*n* = 3), anti-PL-12+ (*n* = 2), and anti-PL-7+ (*n* = 1)] and 39 HCs. Plasma from 25 patients [anti-MDA5+ (*n* = 16), anti-TIF1γ+ (*n* = 5), anti-Jo-1+ (*n* = 3), and MSAs- (*n* = 1)] and 29 HCs were used for cytokine/chemokine quantification. For real-time quantitative polymerase chain reaction (qRT-PCR), the PBMC cDNA was obtained from 93 IIM patients [anti-MDA5+ (*n* = 29) and anti-MDA5- (*n* = 64)] and 57 matched HCs. The monocyte cDNA was obtained from 14 IIM patients [anti-Jo-1+ (*n* = 3), anti-Mi-2+ (*n* = 3), MSAs- (*n* = 3), anti-MDA5+ (*n* = 2), anti-TIF1γ+ (*n* = 2), and anti-PL-7+ (*n* = 1)] and 18 matched HCs. For Western blot analysis, PBMCs were obtained from five patients [anti-Jo-1+ (*n* = 2), MSAs- (*n* = 2), and anti-MDA5+ (*n* = 1)] and eight HCs. Monocytes were obtained from eight patients [anti-MDA5+ (*n* = 2), anti-Jo-1+ (*n* = 2), anti-TIF1γ+ (*n* = 1), and MSAs- (*n* = 3)] and eight HCs. The patients’ clinical characteristics, laboratory parameters, and MSAs detected are shown in **Table 1**.

**Table 1:** Baseline clinical characteristics of idiopathic inflammatory myopathy (IIM) patients in this study.

	RNA sequencing	Validation experiments	
	IIM (*n* = 23)	MDA5 (*n* = 36)	Non-MDA5 (*n* = 99)
Age at baseline, years, mean ± SD	54.91 ± 13.99	51.06 ± 9.43	53.35 ± 12.48
Female, *n* (%)	19 (82.61)	21 (58.33)	78 (78.79)
Disease duration, months, median (interquartile range, IQR)	3 (3–12)	3 (1.17–9)	12 (3–24)
Clinical features, *n* (%)			
Fever	7 (30.43)	15 (41.67)	26 (26.26)
Gottron papules	7 (30.43)	10 (27.78)	13 (13.13)
Gottron sign	2 (8.7)	10 (27.78)	11 (11.11)
Heliotrope rash	13 (56.52)	20 (55.56)	29 (29.29)
Shawl-sign rash	8 (34.78)	10 (27.78)	23 (23.23)
V-sign rash	6 (26.09)	9 (25)	24 (24.24)
Mechanic’s hands	2 (8.7)	4 (11.11)	15 (15.15)
Myalgia	7 (30.43)	14 (38.89)	33 (33.33)
Muscle weakness	8 (34.78)	20 (55.56)	55 (55.56)
Dysphagia	3 (13.04)	4 (11.11)	20 (20.2)
Arthralgia	8 (34.78)	17 (47.22)	33 (33.33)
Raynaud’s phenomenon	2 (8.7)	1 (2.78)	8 (8.08)
Weight loss	1 (4.35)	8 (22.22)	10 (10.1)
Interstitial lung disease	6 (26.09)	22 (61.11)	55 (55.56)
Malignancy	1 (4.35)	0	1 (1.01)
Laboratory features			
WBC count (×10^9^/L), median (IQR)	5.4 (4.4–6.2)	5.41 (4.44–7.7)	6.6 (5.4–8.66) (*n* = 95)
PLT count (×10^9^/L), mean ± SD	212.87 ± 72.12	219.64 ± 78.45	232.91 ± 77.99 (*n* = 94)
Lymphocyte count (×10^9^/L), median (IQR)	1.15 (0.65–1.78)	0.89 (0.51–1.36)	1.33 (0.78–1.84) (*n* = 95)
Hgb (g/L), mean ± SD	118.83 ± 12.7	124.48 ± 13.42	125.33 ± 15.11 (*n* = 95)
ALT (U/L), median (IQR)	61 (13–119) (*n* = 21)	34.5 (17.5–50.5)	22 (12–45) (*n* = 91)
AST (U/L), median (IQR)	59 (19.5–136.5) (*n* = 21)	32.5 (20.25–49)	25.5 (15–52.25) (*n* = 90)
CK (U/L), median (IQR)	115 (27.5–914.25) (*n* = 22)	43 (28.25–98.25)	85 (41–599.5) (*n* = 93)
LDH (U/L), median (IQR)	271 (225.75–479.5) (*n* = 22)	312.5 (219.25–362)	302.5 (223.75–419) (*n* = 94)
ESR (mm/h), median (IQR)	17 (13.5–32) (*n* = 21)	19 (10.25–32.75)	12 (6–22.75) (*n* = 88)
CRP (mg/L), median (IQR)	2.09 (1.25–13.43) (*n* = 21)	1.5 (1.5–9.84)	2.46 (1–11.86) (*n* = 87)
C3 (g/L), mean ± SD	1.02 (0.91–1.17) (*n* = 19)	1.07 ± 0.16 (*n* = 31)	1.02 ± 0.21 (*n* = 85)
C4 (g/L), mean ± SD	0.28 ± 0.11 (*n* = 19)	0.3 (0.24-0.34) (*n* = 32)	0.26 ± 0.08 (*n* = 84)
IgA (g/L), mean ± SD	3.03 ± 1.12 (*n* = 18)	2.87 ± 0.97 (*n* = 29)	2.16 ± 0.96 (*n* = 80)
IgG (g/L), median (IQR)	10.4 (8.65–14.1) (*n* = 18)	11.90 (10.05–13.35) (*n* = 29)	10.9 (8.83–13.5) (*n* = 80)
IgM (g/L), mean ± SD	1 (0.68–1.65) (*n* = 18)	1.07 ± 0.38 (*n* = 29)	1.1 (0.8–2) (*n* = 79)
Ferritin (ng/ml), median (IQR)	462.05 (212.33–734.5) (*n* = 18)	519.25 (305.93–1183.95) (*n* = 34)	228.5 (103.4–355) (*n* = 80)
KL-6 (U/ml), median (IQR)	566 (277–1314.5) (*n* = 21)	747.5 (559.75–1316.75) (*n* = 32)	563 (262–1367) (*n* = 71)
Anti-MDA5 Abs (U/ml), mean ± SD	141.8 ± 57.74 (*n* = 9)	140.7 ± 62.21	0
Myositis autoantibodies, *n* (%)			
MDA5	9 (39.13)	36 (100)	0
Jo-1	4 (17.39)	2 (5.56)	22 (22.22)
TIF1γ	5 (21.74)	1 (2.78)	15 (15.15)
EJ	0	0	10 (10.1)
PL-7	0	1 (2.78)	9 (9.09)
PL-12	0	0	4 (4.04)
Mi-2	0	1 (2.78)	6 (6.06)
NXP-2	0	0	2 (2.02)
SRP	0	1 (2.78)	2 (2.02)
MSAs Neg	5 (21.74)	0	34 (34.34)
Ro52	2 (8.7)	10 (27.78)	42 (42.42)
PM-Scl	0	0	6 (6.06)
Ku	0	0	4 (4.04)
Treatment, *n* (%)			
Prednisone	11 (47.83)	23 (63.89)	67 (67.68)
Immunosuppressive agents	10 (43.48)	16 (44.44)	40 (40.4)
IVIG	3 (13.04)	2 (5.56)	4 (4.04)
Without treatment	7 (30.43)	10 (27.78)	19 (19.19)

qRT-PCR, real-time quantitative polymerase chain reaction; WB, Western blotting; MDA5, anti-melanoma differentiation-associated gene 5 antibody; WBC, white blood cell count; PLT, platelet count; Hgb, hemoglobin; ALT, alanine aminotransferase; AST, aspartate aminotransferase; CK, creatine kinase; LDH, lactate dehydrogenase; ESR, erythrocyte sedimentation rate; CRP, C-reactive protein; C3, complement 3; C4, complement 4; IgA, immunoglobulin A; IgG, immunoglobulin G; IgM, immunoglobulin M; KL-6, Krebs von den Lungen-6; MSAs, myositis-specific antibodies; Jo-1, anti-histidyl-tRNA synthetase antibody; TIF1-γ, anti-transcription intermediary factor-1γ antibody; PL-7, anti-threonyl-tRNA synthetase antibody; PL-12, anti-alanyl-tRNA synthetase antibody; Ku, anti-Ku antibody; EJ, anti-glycyl-tRNA synthetase antibody; PM-Scl, anti-polymyositis scleroderma antibody; Mi-2, anti-Mi-2 antibody; NXP-2, anti-nuclear matrix protein 2 antibody; SRP, anti-signal recognition particle antibody; Ro52, anti-Ro52 antibody; immunosuppressive agents, immunomodulatory drugs include CYC, MTX, AZA, and ciclosporin A; IVIG, intravenous immunoglobulin.

### 2.2 PBMC and monocyte isolation

Peripheral blood (5–10 ml) was collected into EDTA-containing tubes (BD, UK). PBMCs were separated by density gradient centrifugation using Human Lymphocyte Separation Medium (Dakewe, China). The monocytes were isolated from PBMCs using CD14 MicroBeads (Miltenyi Biotec, Germany) according to the manufacturer’s instructions.

### 2.3 RNA-seq analysis

Following the manufacturer’s instructions, total RNA was extracted using mirVana miRNA Isolation Kit (Ambion, USA). Agilent 2100 Bioanalyzer (Agilent Technologies, USA) was utilized to evaluate the RNA integrity. Samples with an RNA integrity number greater than 7 were analyzed further. The libraries were constructed, following the manufacturer’s protocol, with TruSeq Stranded mRNA LTSample Prep Kit (Illumina, USA). These libraries were then sequenced on the Illumina sequencing platform (HiSeqTM 2500 or Illumina HiSeq X Ten), yielding 125/150 bp paired-end reads. Trimmomatic was used to remove ploy N-containing and low-quality reads (23). The clean reads were then mapped to the reference genome with hisat2 (24).

### 2.4 Identification of differentially expressed genes

The “DESeq2” package (version 1.36.0) was used to identify the DEGs between 23 IIM patients and HCs, with the cutoff criteria of |log_2_ fold change| >1 and *p*_adj_ *<*0.05. After rlog transformation, principal component analysis (PCA) was performed using “plotPCA” in “DESeq2” package to identify the clustering of samples. The “ggplot2” and “ComplexHeatmap” packages were used to show the DEGs compared between groups. The top 15 DEGs selected by |log_2_ fold change| were visualized using Cytoscape software (version 3.9.1).

### 2.5 Immune infiltration analysis

To quantify the relative abundance of 22 types of immune cells, we run the CIBERSORT R script (version 1.03) with 1,000 permutations and no quantile normalization. The correlations of immune cells in patients were calculated using Spearman correlation analysis and were visualized with the “corrplot” package (version 0.92). The Wilcoxon test was performed to compare the fraction of immune cells.

### 2.6 Flow cytometry and plasma protein detection

PBMCs were incubated with Human TruStain FcX (BioLegend, USA) and stained with the following antibodies: FITC anti-human CD14 and Alexa Fluor 647 anti-human CD16 (BioLegend, USA). The stained cells were assessed using the FACSCelesta (BD Biosciences, USA). Data were analyzed using FlowJo software (V10.6.2., Tree Star). Nine plasma proteins, including IL-6, IL-8, CSF-1, CCL3, TNF, MCP-1, MCP-2, MCP-3, and MCP-4, were targeted and quantified by Olink multiplex proximity extension assay following the manufacturer’s instructions. The protein abundance levels were reported as normalized protein expression values on a log_2_ scale.

### 2.7 Gene set enrichment analysis

To perform gene set enrichment analysis (GSEA), all genes ranked by log_2_ fold change were analyzed using the “clusterProfiler” package (version 4.4.4), with the reference gene set C2-CP sub-collection (c2.cp.v2022.1.Hs.entrez.gmt) from the Molecular Signatures Database (MSigDB). All or core enriched genes in selected pathways were extracted to calculate the mean value of fragments per kilobase of transcript per million mapped reads (FPKM) for each group and were visualized using the “ComplexHeatmap” package (version 2.12.1) in the form of log_2_(FPKM + 1).

### 2.8 Weighted gene co-expression network analysis

To construct the co-expression network of weighted gene co-expression network analysis (WGCNA), the genes whose counts were less than 10 in more than 90% of the samples were removed. After being standardized by log_2_(FPKM + 1), the top 5,000 genes ordered by median absolute deviation were chosen as input. All 31 samples were analyzed with the “WGCNA” package (version 1.71) in R. The optimal soft-thresholding power was automatically picked as 12 (scale-free *R* ^2 = ^0.8560). The adjacency matrix was used to construct the topological overlap matrix (TOM) and the topological difference matrix (dissTOM) by the dynamic cutting technique. The minimum cluster size = 30, and deepSplit = 2 was set to construct the primary modules. Then, cutHeight was set as 0.25 to merge the modules. In total, 14 merged modules were finally found. The relationships between module eigengenes and traits were assessed by Pearson’s correlation. The traits included the presence or absence of IIM, patient MSA types, clinical features of the disease, and immune cell infiltration results. The top seven modules correlated to the “IIM” trait were selected. After calculating the gene significance (GS) and module membership (MM), the WGCNA hub genes were identified with the criteria of MM >0.8 and GS >0.2. The key IFN genes were identified as the intersection of WGNCA hub genes, DEGs (IIM/HCs), and genes from the Reactome IFN signaling. The protein–protein interaction (PPI) network of the 29 key IFN genes, constructed using the STRING database, was visualized using Cytoscape software (version 3.9.1).

### 2.9 Quantitative real-time polymerase chain reaction

Total RNA was extracted using TRIzol Reagent (Ambion, USA) according to the manufacturer’s instructions. Then, cDNA was obtained using PrimeScript RT Master Mix (Takara, Japan). RNA concentration and quality were determined as measured by NanoDrop One (Thermo Scientific, USA). Quantitative real-time PCR proceeded using TB Green Premix Ex Taq II (Takara, Japan) on Applied Biosystems QuantStudio 3 and 5 (Thermo Fisher Scientific, USA). The *GAPDH* gene was used as an endogenous control. The primer sequences used are listed in **Supplementary Table S1**. The relative expression levels were determined by the 2^-ΔΔCT^ method.

### 2.10 Western blot analysis

Total protein from PBMCs or monocytes was extracted using Total Protein Extraction Kit for Animal Cultured Cells and Tissues (Invent, USA). The protein concentration of each sample was measured with Pierce BCA Protein Assay Kit (Thermo Fisher Scientific, USA). Approximately 10–20 μg of proteins from each sample was separated on FuturePAGE 4-12% 11 Wells (ACE, China) and transferred to 0.45-µm polyvinylidine fluoride (PVDF) membranes (Merck Millipore, Ireland). After being blocked using Quick Block Western blocking solution (Beyotime, China) for 20 min, the PVDF membranes were incubated with primary antibodies (1:1,000 dilution) overnight at 4°C. Then, the membranes were incubated with Goat Anti-Rabbit IgG (H&L)-HRP Conjugated (EASYBIO, BE0101, 1:10,000) for 1 h at room temperature. The membranes were then visualized with Pierce ECL Western Blotting Substrate (Thermo Scientific, USA). Densitometry analysis was performed using Image-J software (NIH). β-Actin was used as an internal control. In the need for stripping and re-probing, the Restore Western Blot Stripping Buffer was used (Thermo Scientific, USA).

The primary antibodies used were Phospho-Stat1 (#9167), Phospho-Stat2 (#88410), Phospho-IRF-7 (#12390), Phospho-SHP-1 (#8849), β-actin (#4970), Stat1(#14995), Stat2 (#72604), IRF-7 (#13014), and SHP-1 (#3759). All primary antibodies used in this study were purchased from Cell Signaling Technology (USA) and used at 1:1,000 dilution.

### 2.11 Statistical analysis and ROC analysis

Statistical analysis and graphing were performed using GraphPad Prism 9 (GraphPad Software, USA) or R (version 4.2.1; https://www.R-project.org/). The data were presented as the average ± standard deviation (SD), and test results are summarized as “ns” for not significant, *p < 0.05, **p < 0.01, ***p < 0.001, and ****p < 0.0001. Statistical significance was considered as *p* < 0.05.

The Wilcoxon test was used to compare the immune cell fractions between groups. The Spearman correlation analysis was performed to investigate the relationship between immune cells and to calculate the correlations between gene expressions and clinical laboratory indicators, including the level of anti-MDA5 Abs. Comparisons between two groups were made using the Welch’s *t*-test, unpaired *t*-test, or Mann Whitney test as appropriate. Kruskal–Wallis test, followed by Dunn’s multiple-comparisons test, was used to compare the three groups.

To assess the performance of distinguishing IIM from HCs, anti-MDA5+ IIM from HCs, or anti-MDA5+ IIMs from anti-MDA5- IIMs, receiver operating characteristic (ROC) curve analyses were performed for each validated gene. The area under the curve (AUC) was calculated with a 95% confidence interval (CI). The ROC analysis results were interpreted as: AUC <0.70, low diagnostic accuracy; 0.70–0.90, moderate diagnostic accuracy; and ≥0.90, high diagnostic accuracy. The ROC curves are shown with *p*-value <0.05.

## 3 Results

### 3.1 Identification of DEGs between IIM and HC

**Figure 1:**
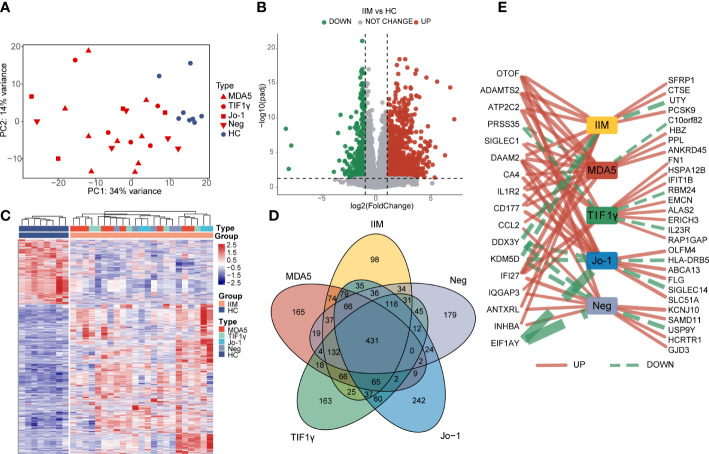
Identification of differentially expressed genes (DEGs) in patients with idiopathic inflammatory myopathies (IIM). **(A)** Principal component analysis (PCA) of transcriptomic profiling in IIM patients with different myositis-specific autoantibodies (*n* = 23) and healthy controls (HCs; *n* = 8). **(B)** Volcano plot showing DEGs between the IIM and HC groups (*p*_adj_ < 0.05, |log_2_ fold change| >1). **(C)** Heat map of DEGs between the IIM and HC groups. **(D)** Venn diagram showing the overlap of DEGs identified by comparing the HC group and patient groups (*p*_adj_ < 0.05, |log_2_ fold change| >1). **(E)** Top15 DEGs identified by comparing the patient groups with HCs. The solid red and dashed green lines represent the up- and downregulated DEGs separately. The increase of the line width means an increase of |log_2_ fold change| from 4.35 to 22.54.

To characterize the gene expressions of IIM patients with different MSA types, we identified the DEGs between HC and IIM or MSA-typed samples (**Figure 1**). From the PCA plot (**Figure 1A**), the transcriptomic profiling of patients with IIM was significantly altered compared with HCs. However, the expression of transcriptomic profiling was narrowed for patients with IIM of different MSAs. A total of 1,364 DEGs were identified in patients with IIM compared with HCs, including 952 upregulated and 412 downregulated genes (**Figure 1B**). The heat map shows the expressions of these DEGs in each sample characterized by MSAs (**Figure 1C**). The Venn diagram shows the overlap of DEGs identified between the HC group and IIM or MSA-typed patients (**Figure 1D**), from which there were some DEGs expressed uniquely in MSA-typed groups, and most of the DEGs were shared in common. The top 15 DEGs identified in different MSA-typed IIM patients are shown in **Figure 1E**.

### 3.2 Landscape of immune cells in the PBMCs of patients with IIM

**Figure 2:**
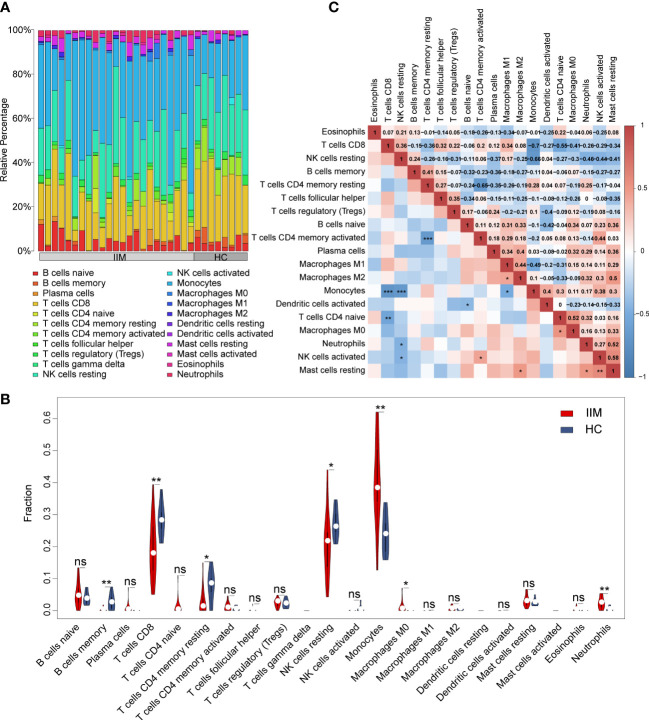
Landscape of immune infiltration in patients with idiopathic inflammatory myopathies (IIM). **(A)** Relative percentage of immune cells identified in patients with IIM (*n* = 23) and healthy controls (HCs; *n* = 8). **(B)** Violin plot showing the comparisons of immune cell fractions between patients with IIM and HCs. ***P < 0.001; **P < 0.01; *P < 0.05; ns, no significance. **(C)** Correlation analyses of immune cells in all patients. ***P < 0.001; **P < 0.01; *P < 0.05.

In order to evaluate the fractions of immune cells in the PBMCs of patients with IIM, CIBERSORTx analysis was performed. By using the CIBERSORT algorithm, the percentages of 22 types of immune cells were quantified in all samples (**Figure 2A**). Compared with HCs, elevated proportions of monocytes, macrophage M0, and neutrophils, as well as decreased proportions of memory B cells, CD8+ T cells, resting memory CD4+ T cells, and resting NK cells, were shown in IIMs (**Figure 2B**). The correlation heat map showed that the monocytes were negatively correlated with CD8+ T cells (*r* = −0.7, *p* < 0.001), resting NK cells (*r* = −0.66, *p* < 0.001), and M1 macrophages (*r* = −0.49, *p* < 0.05) (**Figure 2C**). Immune cell infiltration analysis was also performed in IIM patients with different MSAs (**Supplementary Figures S1A–D**). From the above-mentioned results, the monocyte fractions were significantly increased in the PBMCs of patients with IIM as well as in anti-MDA5+ DM patients, anti-Jo-1+ IIM patients, and MSA- IIM patients.

**Figure 3:**
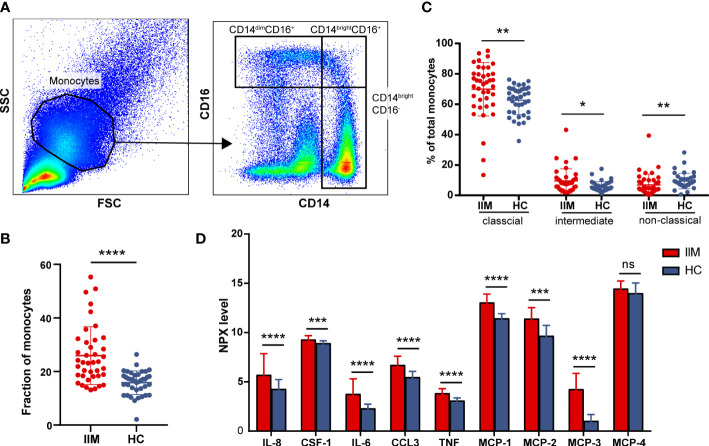
Monocyte subpopulations and plasma protein expressions quantified in idiopathic inflammatory myopathies (IIM). **(A)** Representative flow cytometry gating scheme to identify CD14dimCD16+, CD14brightCD16+, and CD14brightCD16- monocyte subpopulations in peripheral blood mononuclear cells (PBMCs) of IIM patients (*n* = 40) and healthy controls (HCs; *n* = 39). **(B)** Elevated monocyte fractions in PBMCs of IIM patients. **(C)** Changed frequencies of monocyte subpopulations in patients with IIM. **(D)** Expression levels of macrophage–monocyte-related proteins in the plasma of IIM patients (*n* = 25) and HCs (*n* = 29). ****P < 0.0001; ***P < 0.001; **P < 0.01; *P < 0.05; ns, no significance.

To further investigate the potential role of monocytes, we quantified the subsets and related plasma cytokines of monocytes in IIM patients. Three monocyte subpopulations—CD14brightCD16- (classical), CD14brightCD16+ (intermediate), and CD14dimCD16+ (non-classical) monocytes—were quantified within the broad monocyte gate defined by forward and side scatter (**Figure 3A**). The proportion of broad monocytes also increased in patients (25.92% ± 10.78% *vs*. 15.82% ± 4.43%, *p* < 0.0001) identified by flow cytometry, which supported our results in the immune infiltration analysis (**Figure 3B**). At the same time, elevated proportions of classical (69.87% ± 17.58% *vs*. 62.61% ± 9.41%, *p* = 0.0012) and intermediate (9.56% ± 7.90% *vs*. 5.67% ± 3.30%, *p* = 0.0108) populations and decreased non-classical (6.84% ± 7.08% *vs*. 9.41% ± 5.12%, *p* = 0.0018) monocyte subpopulations were identified in IIM patients (**Figure 3C**). Consistent with the monocyte fraction changes analyzed according to MSA types (**Supplementary Figure S2**), the fraction of broad monocytes increased in the PBMCs of anti-MDA5+ (23.36% ± 13.11% *vs*. 15.82% ± 4.43%, *p* = 0.0009), anti-Jo-1+ (29.79% ± 9.82% *vs*. 15.82% ± 4.43%, *p* = 0.0089), and MSA- (21.17% ± 5.08% *vs*. 15.82% ± 4.43%, *p* = 0.0018) IIM patients (**Supplementary Figures S2A–D**). From the monocyte subpopulations quantified in MSA-typed patients (**Supplementary Figures S2E–H**), the percentages of classical (70.21% ± 11.24% *vs*. 62.61% ± 9.41%, *p* = 0.0382) and intermediate (10.32% ± 6.02% *vs*. 5.67% ± 3.30%, *p* = 0.0040) monocyte subpopulations were also elevated in anti-MDA5+ patients; a decreased non-classical (5.12% ± 4.67% *vs*. 9.41% ± 5.12%, *p* = 0.0004) monocyte subpopulation (**Supplementary Figure S2E**) was observed as well. As in **Figure 3D**, the expression levels of monocyte-related cytokines or chemokines also increased in the plasma of patients with IIM, including IL-8 (5.72 ± 2.15 *vs*. 4.28 ± 0.96, *p* < 0.0001), CSF-1 (9.30 ± 0.38 *vs*. 8.93 ± 0.23, *p* = 0.0001), IL-6 (3.78 ± 1.53 *vs*. 2.31 ± 0.43, *p* < 0.0001), CCL3 (6.73 ± 0.88 *vs*. 5.49 ± 0.57, *p* < 0.0001), TNF (3.86 ± 0.45 *vs*. 3.09 ± 0.28, *p* < 0.0001), MCP-1 (13.06 ± 0.84 *vs*. 11.45 ± 0.48, *p* < 0.0001), MCP-2 (11.43 ± 1.10 *vs*. 9.67 ± 1.06, *p* < 0.0001), and MCP-3 (4.27 ± 1.59 *vs*. 1.04 ± 0.64, *p* < 0.0001).

### 3.3 Gene set enrichment analysis identified interferon-related signaling pathways in IIM

**Figure 4:**
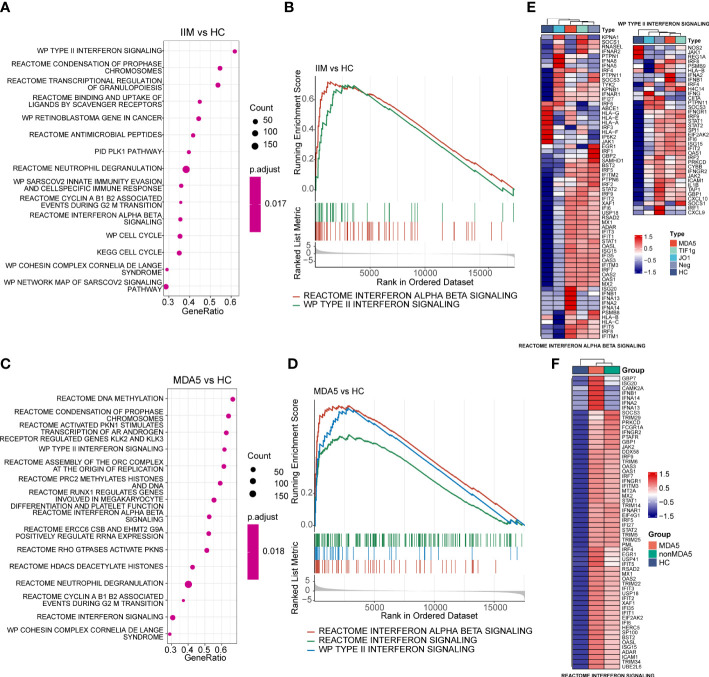
Gene set enrichment analysis of patients with idiopathic inflammatory myopathies (IIM) and anti-MDA5+ dermatomyositis (DM) patients. **(A)** Top 15 significantly enriched pathways in patients with IIM (*n* = 23) compared with healthy controls (HCs; *n* = 8). **(B)** Two interferon (IFN)-related pathways enriched in patients with IIM: reactome interferon alpha beta signaling pathway and WP type II interferon signaling pathway. **(C)** Top 15 significantly enriched pathways in anti-MDA5+ DM patients (*n* = 9) compared with HCs. **(D)** Three IFN-related pathways enriched in the MDA5 group: reactome interferon alpha beta signaling pathway, reactome interferon signaling pathway, and WP type II interferon signaling pathway. **(E)** Gene expression heat map of IFN-related pathways in IIM patients with different myositis-specific autoantibodies. **(F)** Expression pattern of the core enrichment gene in the reactome interferon signaling enriched in anti-MDA5+ DM patients.

To reveal the potential pathogenic pathways involved in IIM, we performed GSEA with all genes and visualized pathway gene expression profiles according to MSAs. The top 15 pathways enriched in patients with IIM are shown in **Figure 4A**. The IFN-I and IFN-II signaling pathways were enriched in the top 15 pathways (**Figure 4B**). Remarkably, three IFN-related pathways were enriched in anti-MDA5+ DM patients (**Figure 4C**), and these three signaling pathways were significantly activated in anti-MDA5+ patients (**Figure 4D**). A pathway enrichment analysis was also performed in patients with IIM of different MSAs (**Supplementary Figures S3A–C**). The results demonstrated that the IFN-I and IFN-II signaling pathways were activated in patients with anti-TIF1-γ antibodies (**Supplementary Figure S3D**) and MSA-negative patients (**Supplementary Figure S3E**). However, differently from patients with other MSAs, only the IFN-I signaling pathway was activated in patients with anti-Jo-1 antibody (**Supplementary Figure S3F**).

The genes in interferon-related pathways were extracted and visualized in heat maps (**Figures 4E, F**). For all genes in these two interferon-related pathways, the patients with different MSAs had distinct expression profiles (**Figure 4E**). The reactome interferon signaling was only enriched in the MDA5 group, and based on the core enriched genes, certain genes like *ISG20*, *IFNA2*, and *IFNB1* were specifically highly expressed in anti-MDA5+ patients (**Figure 4F**). Above all, in contrast with HCs, the activation of interferon-related pathways and the upregulation of IFN-related genes were demonstrated in PBMCs of patients with IIM. The expression of IFN-I and IFN-II related genes was much more prominent in anti-MDA5+ DM patients compared with patients with other MSAs.

### 3.4 Weighted gene co-expression network analysis identified key IFN genes

**Figure 5:**
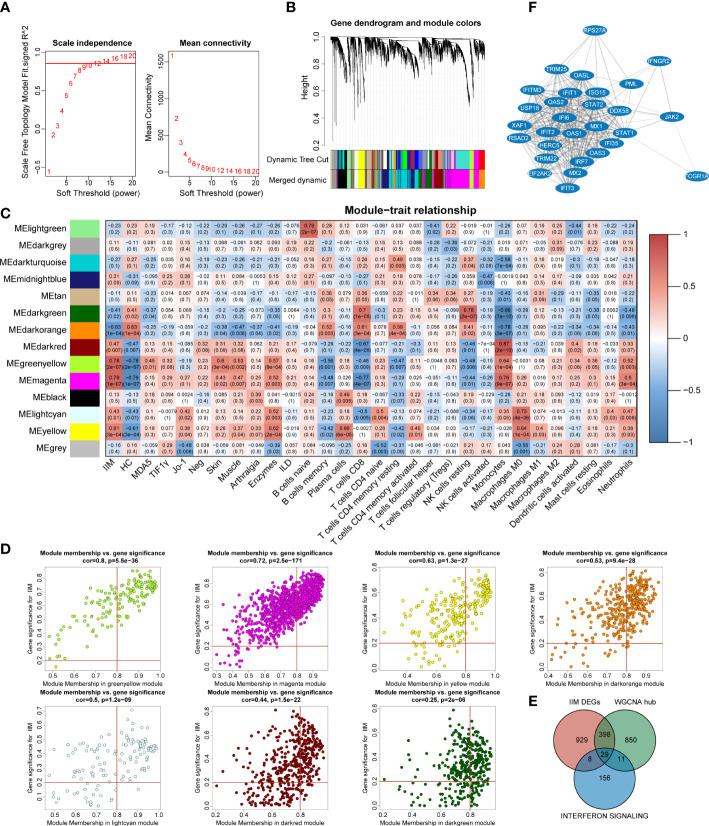
Identification of key interferon-related differentially expressed genes (DEGs) in idiopathic inflammatory myopathies (IIM). **(A)** Analysis of the network topology for selecting the optimal soft threshold power. **(B)** Gene clustering dendrogram and modules merged. **(C)** Correlation between modules and sample traits. **(D)** Scatter plots of gene significance for IIM *vs*. module membership (MM) in yellow-green, magenta, yellow, dark green, dark orange, light cyan, and dark red modules. **(E)** Venn diagram identifying 29 key interferon-related DEGs of IIM. **(F)** Protein–protein interaction network of the 29 key interferon-related DEGs.

To investigate the potential key genes in IIM, we performed WGCNA analysis to construct gene co-expression networks, identified gene modules related to clinical features, and found out the key DEGs involved in IIM interferon signaling. The optimal soft threshold value was selected as 12 (scale-free *R* ^2^ = 0.8560) to establish a scale-free network (**Figure 5A**). A total of 14 merged gene modules were obtained (**Figure 5B**). Traits including the presence or absence of IIM, autoantibody type of patients, clinical features of the disease, and immune infiltration of samples were used to identify key gene modules. Based on the heat map, the overall IIM trait had closer relationships with the modules than each autoantibody-typed IIM trait (**Figure 5C**).

Thus, we selected seven modules based on their eigengene correlations with the IIM trait for further analysis: magenta (*r* = 0.79, *p* = 1e-07), yellow-green (*r* = 0.78, *p* = 2e-07), dark orange (*r* = -0.63, *p* = 1e-04), yellow (*r* = 0.61, *p* = 3e-04), dark red (*r* = 0.47, *p* = 0.007), light cyan (*r* = 0.43, *p* = 0.01), and dark green (*r* = -0.41, *p* = 0.02) modules. At the same time, these modules also showed close relationships with other traits (**Figure 5C**). For the IIM trait, a total of 1,288 hub genes were identified in the seven modules with the following criteria: |GS| >0.2 and |MM| >0.8 (**Figure 5D**).

To further identify significant genes involved in interferon signaling pathways, a conjoint analysis was performed with the DEGs of IIM, hub genes identified in WCGNA, and 204 genes from the reactome IFN signaling. A total of 29 genes were recognized as overlapped genes (**Figure 5E**), and a PPI network of these genes was constructed to demonstrate their relationships, which contained *TRIM25*, *DDX58*, *IRF7*, *STAT1*, *STAT2*, and so on (**Figure 5F**). Moreover, *IFIH1*, *ADAR*, and, *TBX21* were identified as hub genes, which were also related to IFN pathways.

### 3.5 Validation of IFN-related genes in the PBMCs of patients with IIM

To validate our findings, we further evaluated the interested IFN-related genes in IIM patients. *TRIM25*, *DDX58*, *IRF7*, *STAT1*, and *STAT2* genes were chosen from among the 29 common genes. *IFIH1*, *ADAR*, and *TBX21* were selected from WGCNA hub genes, in which *IFIH1* was also from IIM DEGs and *ADAR* belonged to the reactome interferon signaling. We also validated *MNDA* from the DEGs of patients with IIM.

**Figure 6:**
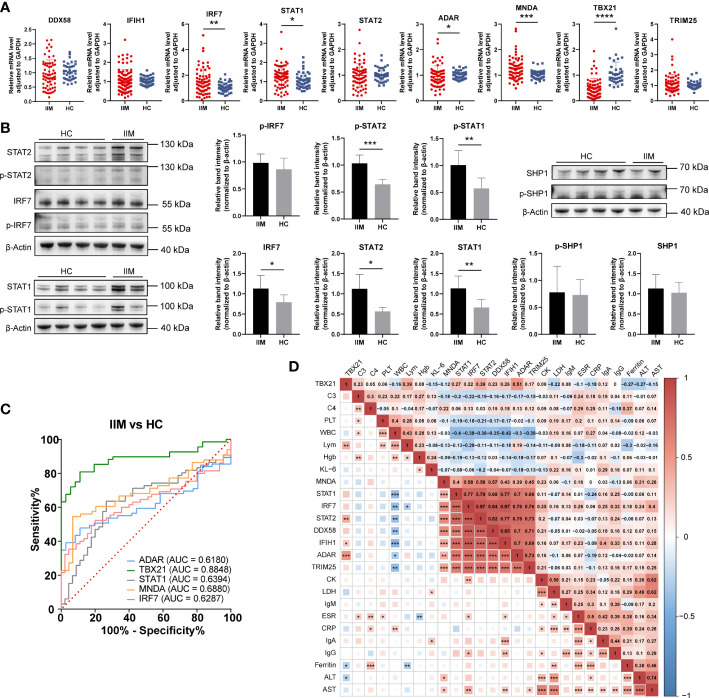
Validation of interferon-related genes in the peripheral blood mononuclear cells of patients with idiopathic inflammatory myopathies (IIM). **(A)** Expression levels of *DDX58*, *IFIH1*, *IRF7*, *STAT1*, *STAT2*, *ADAR*, *MNDA*, *TBX21*, and *TRIM25* detected in patients with IIM (*n* = 93) and heathy controls (HCs; *n* = 57) by qRT-PCR. **(B)** Expression of IRF7, p-IRF7, STAT1, p-STAT1, STAT2, p- STAT2, SHP1, and p-SHP1 detected in patients with IIM (*n* = 5) and HCs (*n* = 8) by Western blotting. **(C)** Receiver operating characteristic analyses of the validated genes in the diagnosis of IIM. **(D)** Heat map of the correlation between laboratory parameters and gene expressions in patients with IIM. *p < 0.05, **p < 0.01, ***p < 0.001, and ****p < 0.0001.

As shown in **Figure 6A**, the upregulation of *IRF7* (1.51 ± 0.99 *vs*. 1.06 ± 0.36, *p* = 0.0044), *STAT1* (1.27 ± 0.58 *vs*. 1.06 ± 0.39, *p* = 0.0126), and *MNDA* (1.27 ± 0.44 *vs*. 1.02 ± 0.17, *p* = 0.0008) and the downregulation of *ADAR* (0.95 ± 0.41 *vs*. 1.01 ± 0.16, *p* = 0.0480) and *TBX21* (0.49 ± 0.38 *vs*. 1.07 ± 0.44, *p* < 0.0001) were detected in patients with IIM compared with HCs. We also detected the levels of IRF7, STAT1, STAT2, and SHP-1 proteins. As shown in **Figure 6B**, the increased protein expression of IRF7 (1.13 ± 0.32 *vs*. 0.79 ± 0.18, *p* = 0.0336), STAT1 (1.14 ± 0.30 *vs*. 0.66 ± 0.20, *p* = 0.0055), STAT2 (1.12 ± 0.36 *vs*. 0.56 ± 0.10, *p* = 0.0234), p-STAT1 (1.00 ± 0.27 *vs*. 0.57 ± 0.19, *p* = 0.0063), and p-STAT2 (1.03 ± 1.16 *vs*. 0.64 ± 0.09, *p* = 0.0001) was shown in the PBMCs of patients with IIM, and this altered protein expression mode represents the activation of IFN-I signaling and downstream JAK–STAT pathways. To further investigate the clinical meaning of these nine validated genes, we performed ROC analyses and calculated correlations between their mRNA expression and clinical laboratory data. As in **Figure 6C**, *TBX21* (AUC = 0.8848, 95%CI = 0.8206–0.9490) had a moderate value in IIM diagnosing, and *ADAR* (AUC = 0.6180, 95%CI = 0.5135–0.7224), *STAT1* (AUC = 0.6394, 95%CI = 0.5346–0.7442), *MNDA* (AUC = 0.6880, 95%CI = 0.5889–0.7872), and *IRF7* (AUC = 0.6287, 95%CI = 0.5251–0.7323) had a low diagnostic value. Their expressions were also related to some clinical indicators, such as WBC, ESR, AST, and so on (**Figure 6D**).

**Figure 7:**
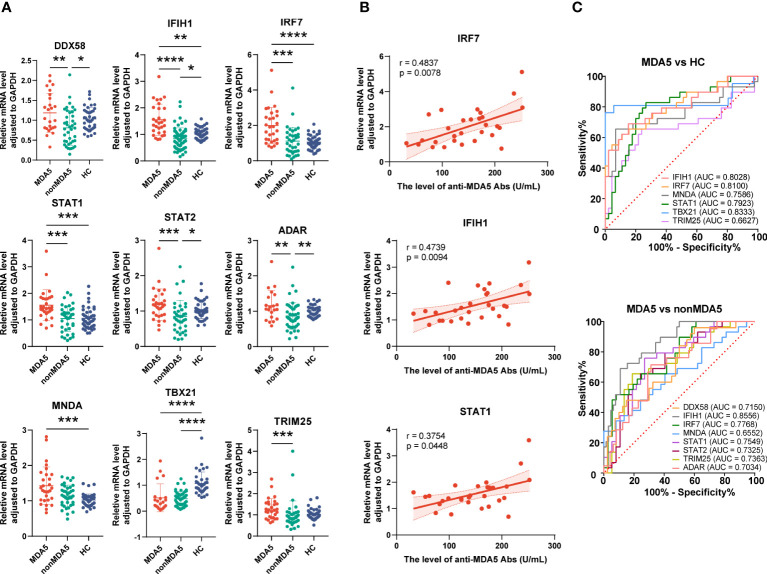
Expression of interferon-related genes in the peripheral blood mononuclear cells of anti-MDA5+ idiopathic inflammatory myopathy (IIM) patients. **(A)** The expression levels of the nine validated genes were compared among anti-MDA5+ IIM patients (*n* = 29), anti-MDA5- IIM patients (*n* = 64), and healthy controls (*n* = 57). **(B)** Correlation analyses of anti-MDA5 antibody levels with the expressions of *IFIH1*, *IRF7*, and *STAT1* genes in anti-MDA5+ IIM patients, with the correlation coefficient *r* values, *p* values, and 95% confidence intervals indicated. **(C)** Receiver operating characteristic analyses of the validated genes in distinguishing anti-MDA5+ patients. *p < 0.05, **p < 0.01, ***p < 0.001, and ****p < 0.0001.

To know the expressions of the validated genes in anti-MDA5+ patients, comparisons were also made within IIM patients. Increased levels of *IFIH1* (1.55 ± 0.59 *vs*. 1.01 ± 0.21, *p* = 0.0011), *IRF7* (1.99 ± 1.02 *vs*. 1.06 ± 0.36, *p* < 0.0001), *STAT1* (1.56 ± 0.58 *vs*. 1.06 ± 0.39, *p* = 0.0001), and *MNDA* (1.43 ± 0.53 *vs*. 1.02 ± 0.17, *p* = 0.0003) as well as decreased levels of *TBX21* (0.52 ± 0.54 *vs*. 1.07 ± 0.44, *p* < 0.0001) were shown in anti-MDA5+ IIM patients in contrast with HCs (**Figure 7A**). Compared with patients without anti-MDA5 antibodies, elevated expressions of *DDX58* (1.19 ± 0.49 *vs*. 0.81 ± 0.44, *p* = 0.0056), *IFIH1* (1.55 ± 0.59 *vs*. 0.84 ± 0.41, *p* < 0.0001), *IRF7* (1.99 ± 1.02 *vs*. 1.11 ± 0.79, *p* = 0.0001), *STAT1* (1.56 ± 0.58 *vs*. 1.05 ± 0.48, *p* = 0.0008), *STAT2* (1.18 ± 0.45 *vs*. 0.85 ± 0.46, *p* = 0.0008), *ADAR* (1.16 ± 0.43 *vs*. 0.86 ± 0.38, *p* = 0.0072), and *TRIM25* (1.28 ± 0.48 *vs*. 0.99 ± 0.67, *p* = 0.0005) were found in anti-MDA5+ IIM patients (**Figure 7A**). Moreover, for anti-MDA5+ IIM patients, correlation analyses of the levels of anti-MDA5 Abs with IFN-I genes were performed. The levels of anti-MDA5 Abs correlated positively with *IRF7* (*r* = 0.4837, 95%CI = 0.1314–0.7276, *p* = 0.0078), *IFIH1* (*r* = 0.4739, 95%CI = 0.1188–0.7215, *p* = 0.0094), and *STAT1* (*r* = 0.3754, 95%CI = -0.0011–0.6586, *p* = 0.0448) (**Figure 7B**), while there was no correlation between the levels of anti-MDA5 Abs with *DDX58*, *STAT2*, *ADAR*, *MNDA*, *TBX21*, and *TRIM25*. For anti-MDA5+ IIM diagnosis, *IFIH1* (AUC = 0.8028, 95%CI = 0.6944–0.9113), *IRF7* (AUC = 0.8100, 95%CI = 0.7053–0.9146), *MNDA* (AUC = 0.7586, 95%CI = 0.6317–0.8855), *STAT1* (AUC = 0.7923, 95%CI = 0.6860–0.8987), and *TBX21* (AUC = 0.8333, 95%CI = 0.6870–0.9796) had a moderate potential, and *TRIM25* (AUC = 0.6627, 95%CI = 0.5220–0.8033) had a low potential (**Figure 7C**). Eight of the nine validated genes could distinguish anti-MDA5+ IIM patients from anti-MDA5- patients, and most of them showed a moderately distinguished value (**Figure 7C**).

From the discussion above, genes related to IFN pathway were highly expressed in patients with IIM, at both mRNA and protein levels. These IFN-related genes also had the potential in disease diagnosis and were correlated with multiple laboratory parameters, indicating their clinical significance for IIM. Moreover, compared with anti-MDA5- patients with IIM, a more predominant IFN gene signature was shown in anti-MDA5+ IIM patients, indicating the pathogenetic difference of patients with different MSAs.

### 3.6 Expression of IFN-related genes in monocytes of patients with IIM

**Figure 8:**
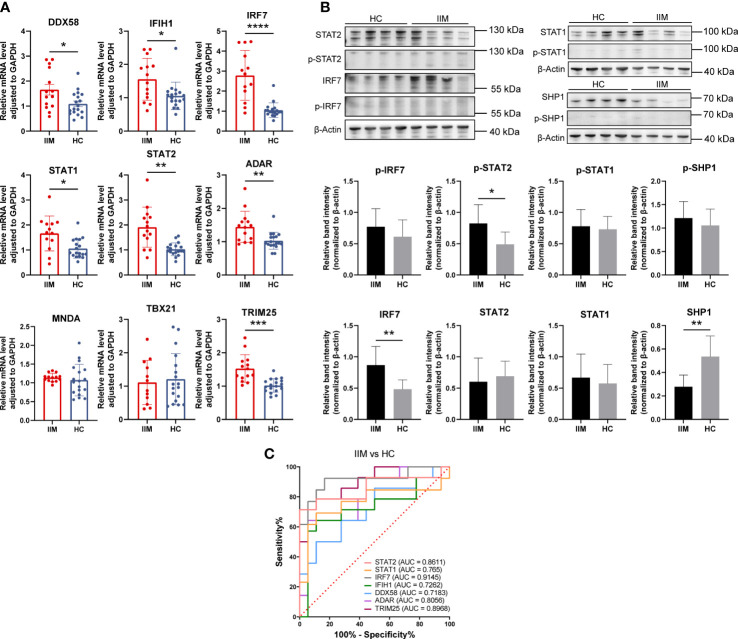
Validation of interferon-related genes in the monocytes of patients with idiopathic inflammatory myopathies (IIM). **(A)** Expression levels of *DDX58*, *IFIH1*, *IRF7*, *STAT1*, *STAT2*, *ADAR*, *MNDA*, *TBX21*, and *TRIM25* detected in the monocytes of patients with IIM (*n* = 14) and healthy controls (HCs; *n* = 18) by qRT-PCR. **(B)** Expression of IRF7, p-IRF7, STAT1, p-STAT1, STAT2, p-STAT2, SHP1, and p-SHP1 detected in the monocytes of patients with IIM (*n* = 8) and HCs (*n* = 8) by Western blotting. **(C)** Receiver operating characteristic analyses of the genes expressed by monocytes in the diagnosis of IIM. *p < 0.05, **p < 0.01, ***p < 0.001, and ****p < 0.0001.

As we have demonstrated above, monocytes and monocyte subpopulations were remarkably altered in the PBMCs of patients with IIM. The levels of monocyte related cytokines were increased as well (**Figure 3**). Next, IFN-related gene expression was then quantified in monocytes. The results revealed that *DDX58* (1.66 ± 0.80 *vs*. 1.09 ± 0.48, *p* = 0.0294), *IFIH1* (1.56 ± 0.63 *vs*. 1.06 ± 0.41, *p* = 0.0304), *IRF7* (2.78 ± 1.25 *vs*. 1.05 ± 0.37, *p* < 0.0001), *STAT1* (1.67 ± 0.70 *vs*. 1.06 ± 0.39, *p* = 0.0116), *STAT2* (1.91 ± 0.80 *vs*. 1.03 ± 0.26, *p* = 0.0013), *ADAR* (1.44 ± 0.47 *vs*. 1.03 ± 0.25, *p* = 0.0027), and *TRIM25* (1.53 ± 0.41 *vs*. 1.02 ± 0.20, *p* = 0.0005) were elevated in the monocytes of patients with IIM (**Figure 8A**). Increased protein levels of IRF7 (0.87 ± 0.30 *vs*. 0.48 ± 0.15, *p* = 0.0058) and p-STAT2 (0.82 ± 0.30 *vs*. 0.49 ± 0.20, *p* = 0.0198) and decreased SHP1 (0.28 ± 0.10 *vs*. 0.54 ± 0.18, *p* = 0.0029) levels were also shown (**Figure 8B**). The ROC analysis indicated that *IRF7* (AUC = 0.9145, 95%CI = 0.8001–1.0000) expressed by monocytes had a high accuracy in the diagnosis of IIM (**Figure 8C**), and monocyte-expressed *STAT1* (AUC = 0.7650, 95%CI = 0.5672–0.9627), *STAT2* (AUC = 0.8611, 95%CI = 0.7115–1.0000), *IFIH1* (AUC = 0.7262, 95%CI = 0.5314–0.9209), *DDX58* (AUC = 0.7183, 95%CI = 0.5348–0.9017), *ADAR* (AUC = 0.8056, 95%CI = 0.6510–0.9601), and *TRIM25* (AUC = 0.8968, 95%CI = 0.7895–1.0000) demonstrated a moderate value in the diagnosis of patients with IIM (**Figure 8C**).

From these results, IFN-related genes were highly expressed by monocytes of IIM patients. Combined with its elevated subpopulations and related cytokine levels, monocytes may play an important role in IIM disease.

## 4 Discussion

Our study shows that gene expression profiles are significantly altered in patients with IIM, including the activation of IFN signaling. IFN-related genes were more prominently expressed in anti-MDA5+ IIM patients compared with patients with other MSAs. Monocytes rose in patients, exhibited a proinflammatory feature, and contributed to IFN signature.

Interferons are cytokines that can be widely produced by cells to induce antiviral states and regulate immune responses when the human body is invaded by pathogens (25). Type I interferon (IFN-I), type II interferon (IFN-II), and type III interferon (IFN-III) share overlapping downstream pathways like the JAK–STAT signaling pathway (26). Aberrant IFN signatures, especially IFN-I, have been reported in IIM. As described before, an activated IFN signature has been verified in diverse tissue types (11–13). The IFN signature also differs in clinical phenotypic IIM subsets, such as activated IFN pathways in the muscles of adult DM patients, instead of in others like PM (10, 27). Moreover, distinct expressions of IFN1-inducible and IFN2-inducible genes were also observed in the muscles of patients (27). In DEG identification of this study, MSA-typed IIM patients shared most of the overlapped DEGs, with some being uniquely expressed, where *IFI27* (*ISG12*), identified as a commonly upregulated topmost DEG in patients, was reported to be transcriptionally upregulated in response to IFN-I (28). Pathway enrichment analysis revealed the consistent activation of IFN-related pathways. More topmost IFN-related genes were enriched in anti-MDA5+ patients, while anti-Jo-1+ patients had fewer enriched IFN-related pathways. Just as in a study by Iago Pinal-Fernandez et al., IFN-I inducible genes were highly expressed in DM and moderately expressed in anti-synthetase syndrome (27).

We verified nine IFN-related genes in an expanded patient cohort. At both mRNA and protein levels, our validation confirms the IFN signaling activation in IIM PBMCs, and the genes involved are helpful in disease diagnosis. Anti-MDA5+ patients had a more pronounced degree of IFN-related gene deregulation than anti-MDA5- patients. Previous studies have pointed out the key role of IFN pathways in IIM pathophysiology (10)—for instance, IFN-I disrupts myoblast differentiation and induces myotube atrophy *in vitro* as well as undermines vascular network organization (29). Mitochondrial dysfunctions mediated by IFN-β-induced ROS lead to muscle inflammation and thus can cause a disease to be self-sustaining (28). Accumulating evidence indicates the possible correlation of MSAs and IFN in IIM. In an experimental myositis model induced with TIF1γ, IFN-I is essential (30). The TRIM33/TIF1γ deficiency results in a high and sustained expression of interferon-β gene in macrophages (31). Immune complexes (ICs) containing anti-Jo-1 and RNA may act as endogenous inducers to activate IFN-α production (32). In muscle tissues from anti-Jo-1+ IIM patients, the B-cell-activating factor of the tumor necrosis factor family (BAFF) is involved in autoantibody production, of which the levels may be influenced by IFN-I (33). Our work complements the previous studies by characterizing the gene expression profiles of PBMCs in IIM patients with different MSAs. Our finding supports the clinical treatment consideration targeted by the IFN pathway, including the use of anti-IFN-α antibody sifalimumab and the use of JAK inhibitors like ruxolitinib and tofacitinib (29, 34, 35).

Upregulated IFN signatures in anti-MDA5+ IIM patients have been reported, especially when compared with anti-MDA5- patients. The serum IFN-α of anti-MDA5+ patients can be used as a biomarker and may reflect the existence of a rapidly progressive interstitial lung disease (36). A stronger IFN-I signature was found in the skin tissue of anti-MDA5+ than anti-MDA5- DM, and IFN-κ, mainly secreted by keratinocytes, possibly participates in skin pathophysiology (37). The expressions of ISGs were also upregulated in a muscle biopsy of anti-MDA5+ DM; however, the IFN score was lower than in classic DM patients (38). Our research shows that the PBMCs of anti-MDA5+ IIM patients have a more pronounced IFN signature, which is correlated positively with the level of anti-MDA5 Abs. This observation may be explained by many findings that highlighted the correlations of anti-MDA5 antibodies (Abs) and IFN. The MDA5 protein is a viral dsRNA sensor which can induce antiviral gene transcription like IFN-I genes and promote proinflammatory cytokine production (39). Abs may bind to MDA5+ cells to induce the aberrant activation of IFN pathway (39). ICs formed by MDA5 and Abs induce IFN-α production *in vitro*, and other monoclonal autoantibodies that existed in anti-MDA5+ DM patients could trigger IFN-γ production directly (40, 41). Combined with previous evidence, our findings strongly support the anti-IFN treatment choice for IIM patients with anti-MDA5 antibody. Additionally, pathways like neutrophil granulation and DNA methylation were also highly activated in anti-MDA5+ patients. Methylation alterations have been found out in affected muscles of JDM, which relate to a self-renewal capacity (42). The aberrant DNA methylation in CD4+ T cells was also found to be associated with systemic lupus erythematosus (SLE) and systemic sclerosis (43).

The monocytes in our study fill a gap of previous studies showing the proinflammatory role of monocytes in IIM. Both from immune cell infiltration and flow cytometry results, the monocytes increased. Patients with IIM had more classical and intermediate monocyte subpopulations and also fewer non-classical subpopulations. This change was also manifested by anti-MDA5+ patients. The plasma levels of monocyte-related proinflammatory cytokines and chemokines increased, and the monocytes in patients had upregulated IFN-related gene expressions. Consistent with a previous research, patients with IIM had increased monocytes (44). Active IIM patients were found with decreased classical and increased intermediate monocyte subsets, and intermediate monocytes increased in treatment-responsive patients, while it decreased in non-responders (45, 46). Our data showed elevated classical and intermediate as well as reduced non-classical subset fractions of monocytes, representing more proinflammatory and fewer anti-inflammatory phenotypic monocytes in IIM. Monocytes/macrophages have been verified as the major producer of inflammatory cytokines in the arthritic lesions of rheumatoid arthritis (47). We also found the plasma level of proinflammatory cytokines like IL-6 and TNF as well as chemokines like CCL3 and MCPs to have been increased, suggesting the possibly promoted proinflammatory cytokine secretion by monocytes and enhanced chemotaxis to damaged sites. The monocytes in patients also expressed upregulated IFN-related genes, which, with its increased amount, may be a potentially significant cell source of IFN signatures. The monocytes/macrophages have been found to be associated with the IFN signature of PBMCs of SLE, in which the classical subset is the primary IFN-I responder (48). Here in this research, we find that the monocytes of IIM patients exhibited a proinflammatory characteristic, including increase to a broad population and altered subset fractions, and possibly promoted cytokine/chemokine production, which also contribute to the IFN signature. In the study of Ye et al., the immune signatures of peripheral B and T cells were demonstrated, revealing the IFN-I signature in anti-MDA5+ DM patients (14). We investigated the transcriptomic profiling of IIM patients with different MSAs in our study. The IFN-I and IFN-II signatures were prominent in anti-MDA5+ IIM patients compared with patients with other MSAs. In addition, we emphasized the pivotal role of monocytes in patients with IIM. Monocytes exhibited a proinflammatory feature and contributed to the IFN signature of IIM patients in our study.

## 5 Limitations

This study had several limitations. Firstly, patients with IIM of all kinds of MSAs were not enrolled. Secondly, this was a single-center study. Thirdly, absolute count beads were not added in performing the flow cytometry experiment, so it was not able to calculate the absolute counts of monocytes in our study. Fourthly, the mechanisms and factors influencing the activation of the IFN pathway in patients with anti-MDA5+ IIM need to be further explored. Furthermore, the proinflammatory role of monocytes with IFN-I activation in the pathogenesis of anti-MDA5+ IIM patients is worth exploring.

## 6 Conclusions

In conclusion, our study demonstrated that the genes in patients with IIM were remarkably altered compared with HCs. IFN signatures were found in IIM patients with different MSAs. IFN expression profiling was more prominent in anti-MDA5+ DM patients. The monocytes showed a proinflammatory characteristic and contributed to the IFN signature of PBMCs of IIM patients.

## Data availability statement

The data presented in the study are deposited in the NCBI's Sequence Read Archive (SRA) repository, accession number PRJNA960687.

## Ethics statement

The studies involving human participants were reviewed and approved by the Ethics Committee of the First Affiliated Hospital of Zhengzhou University. The patients/participants provided their written informed consent to participate in this study.

## Author contributions

Study design: PZ, SL, and ML. Sample collection: PZ, ML, YZ, JS, ZP, and SL. Sample processing, experimental validation, and data analysis: ML, PZ, and YS. Drafting of the article: ML. Review and editing: PZ, YZ, WZ, RL, and SL. All authors contributed to the article and approved the submitted version.
