# Supplementary material for: Type 1 interferon signature in peripheral blood mononuclear cells and monocytes of idiopathic inflammatory myopathy patients with different myositis-specific autoantibodies
Source: Front Immunol. 2023 May 9;14:1169057. doi: 10.3389/fimmu.2023.1169057 (PMC10203462; doi:10.3389/fimmu.2023.1169057)
Supplement: Supplementary file 1 [file DataSheet_1.pdf]

Supplementary Material

Type 1 interferon signature in peripheral blood mononuclear cells and monocytes of idiopathic inflammatory myopathy patients with different myositis-specific autoantibodies

Mengdi Li, Yusheng Zhang, Wenzhe Zhang, Jinlei Sun, Rui Liu, Zhou Pan, Panpan Zhang*, Shengyun Liu*

*** Correspondence:**

Panpan Zhang: panpanzhang2016@163.com

Shengyun Liu: fccliusy2@zzu.edu.cn

1 Supplementary Figures

1.1 Supplementary Figure 1.

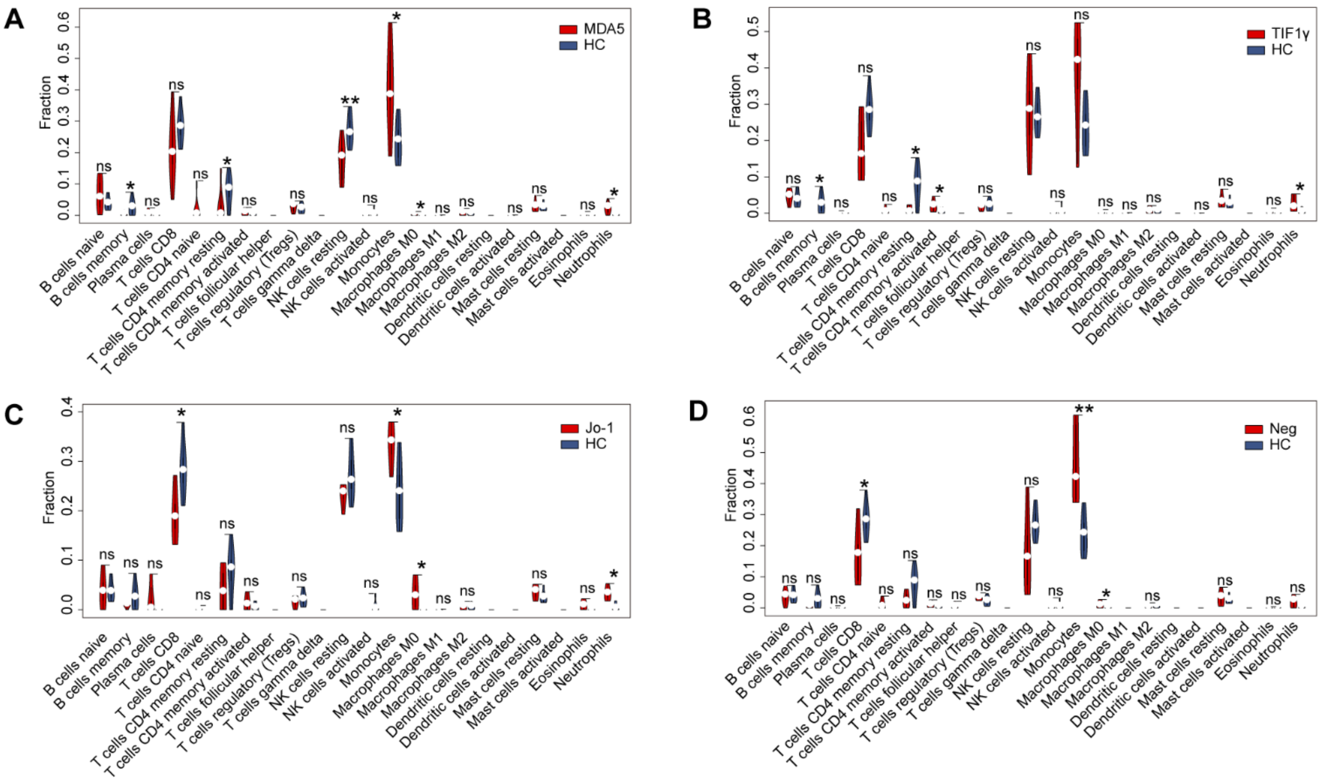

Figure S1. The comparisons of immune cell fractions in patients with IIM of different MSAs. Immune cell fractions compared between HCs (n = 8) and anti-MDA5+ IIM patients (n = 9) (A), patients with anti-TIF1γ antibodies (n = 5) (B), patients with anti-Jo-1 antibodies (n = 4) (C), and patients without MSAs (n = 5) (D). **** P < 0.0001; *** P < 0.001; ** P < 0.01; * P < 0.05; ns no-significance.

1.2 Supplementary Figure 2.

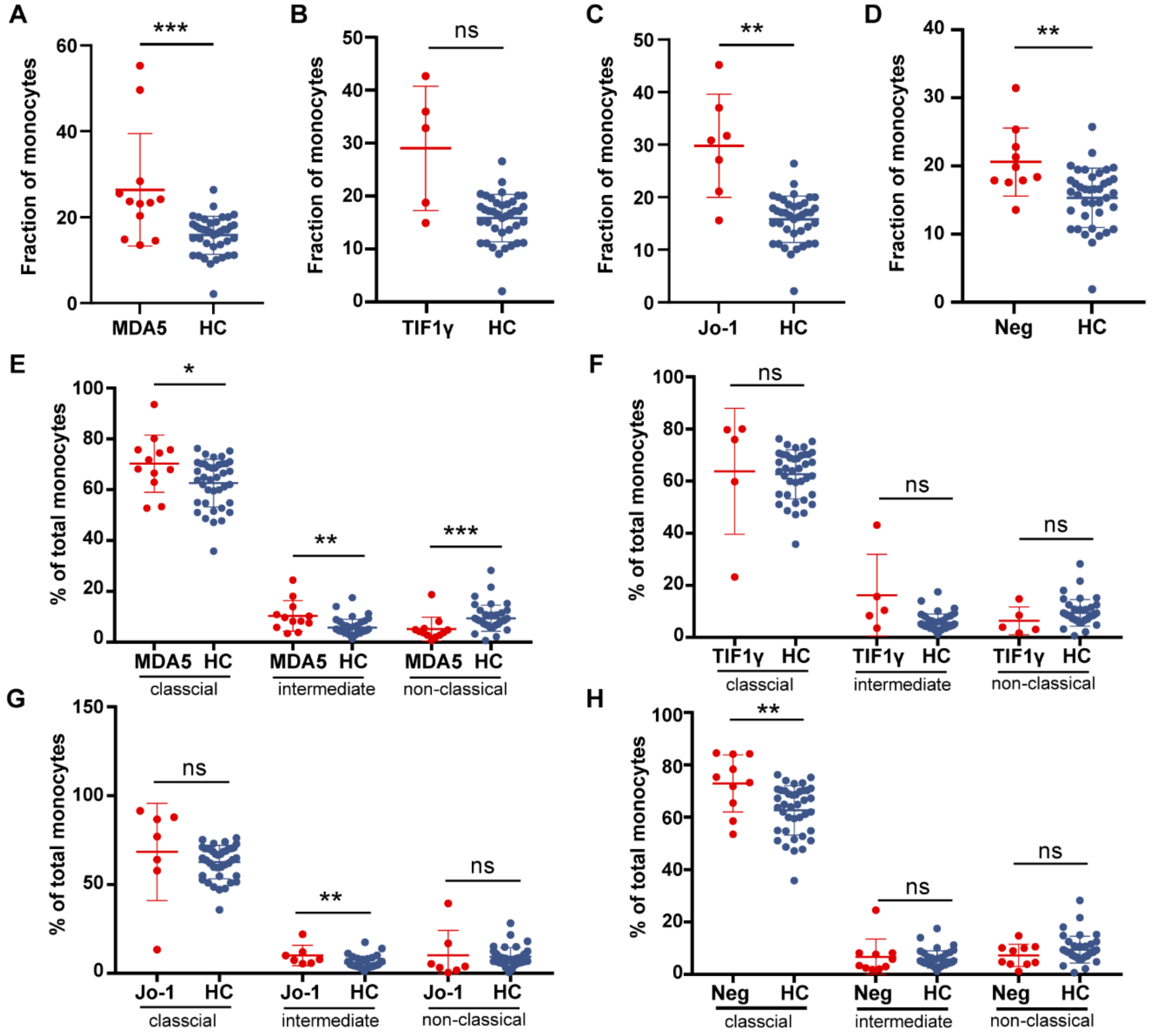

Figure S2. Fraction changes of monocyte subsets quantified in MSA-typed IIM patients. Broad monocyte fractions compared between HCs (*n* = 39) and anti-MDA5+ IIM patients (*n* = 12) (A), patients with anti-TIF1γ antibodies (*n* = 5) (B), patients with anti-Jo-1 antibodies (*n* = 7) (C), and patients without MSAs (*n* = 10) (D). Monocyte subset changes in anti-MDA5+ IIM patients (E), patients with anti-TIF1γ antibodies (F), patients with anti-Jo-1 antibodies (G), and patients without MSAs (H). **** P < 0.0001; *** P < 0.001; ** P < 0.01; * P < 0.05; ns no-significance.

1.3 Supplementary Figure 3.

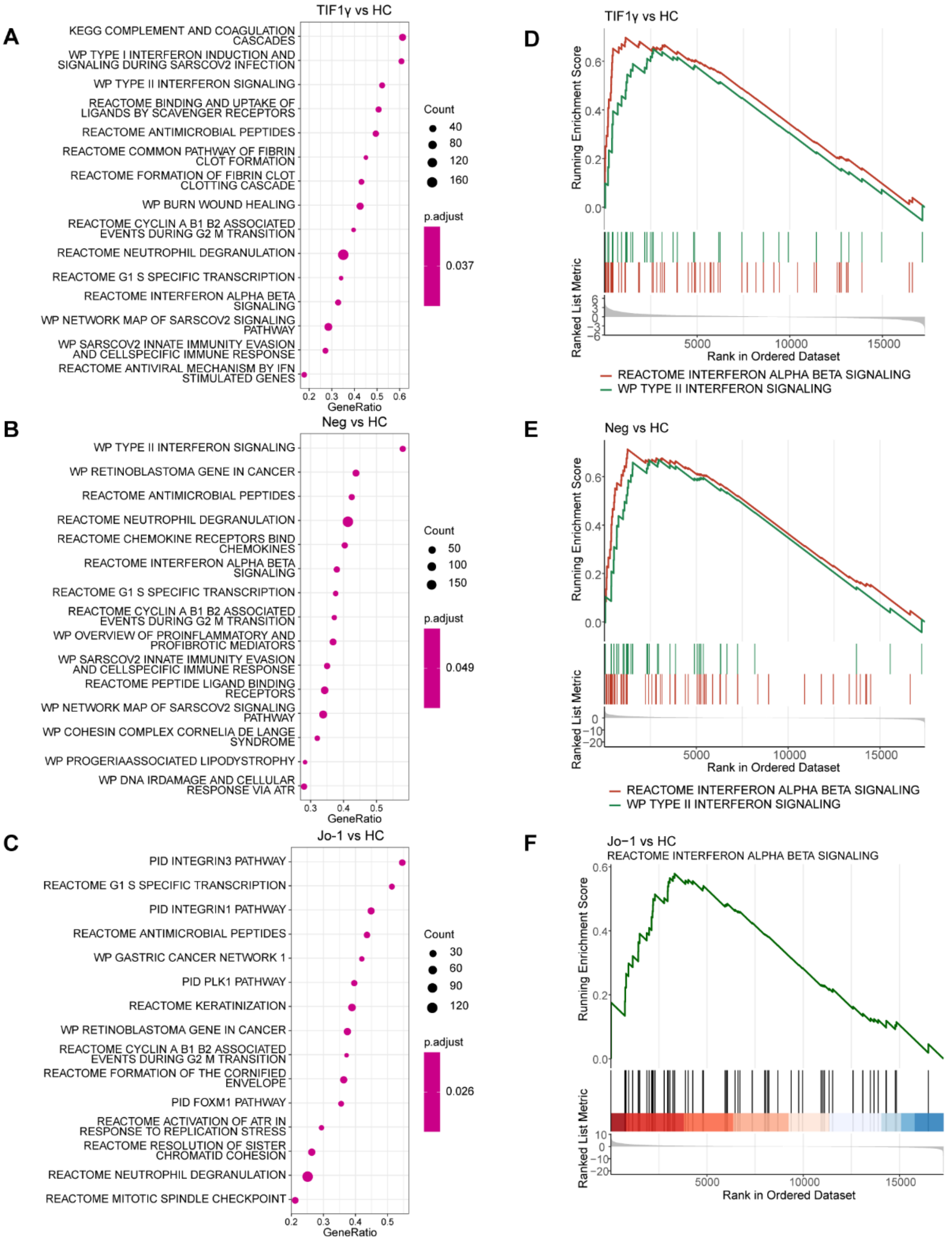

Figure S3. The top 15 significantly enriched pathways in patients with IIM of different MSAs. (A) The top 15 pathways enriched in patients with anti-TIF1γ antibodies (*n* = 5). (B) The top 15 pathways enriched in patients without MSAs (*n* = 5). (C) The top 15 pathways enriched in patients with anti-Jo-1 antibodies (*n* = 4). (D) Two IFN-related pathways of the top 15 upregulated in patients with anti-TIF1γ antibodies. (E) Two IFN-related pathways of the top 15 upregulated in patients without MSAs. (F) The IFN-I pathway was upregulated in patients with anti-Jo-1.

2 Supplementary Table

2.1 Supplementary Table 1.

Gene	Direction	Sequence (5'->3')
DDX58	Forward primer	TGTGCTCCTACAGGTTGTGGA
	Reverse primer	CACTGGGATCTGATTCGCAAAA
IFIH1	Forward primer	TCGAATGGGTATTCCACAGACG
	Reverse primer	GTGGCGACTGTCCTCTGAA
TRIM25	Forward primer	AGGGATGAGTTCGAGTTTCTGG
	Reverse primer	GTTTTTGAGGTCTATGGTGCTCT
IRF7	Forward primer	GCTGGACGTGACCATCATGTA
	Reverse primer	GGGCCGTATAGGAACGTGC
STAT1	Forward primer	ATCAGGCTCAGTCGGGGAATA
	Reverse primer	TGGTCTCGTGTTCTCTGTTCT
STAT2	Forward primer	GAGCCAGCAACATGAGATTGA
	Reverse primer	GCCTGGATCTTATATCGGAAGCA
MNDA	Forward primer	GTTTACTCCGAATCAGGAAACCC
	Reverse primer	TAAATGGCGCTGTTGCTTTCA
GAPDH	Forward primer	TCAACGACCACTTTGTCAAGCTCA
	Reverse primer	GCTGGTGGTCCAGGGGTCTTACT
TBX21	Forward primer	GGATGCGCCAGGAAGTTTCA
	Reverse primer	TGGAGCACAATCATCTGGGT
ADAR	Forward primer	CTGAGACCAAAAGAAACGCAGA
	Reverse primer	GCCATTGTAATGAACAGGTGGTT

Table S1. Primer sequences of genes used for qRT-PCR.
